# Patient-reported outcomes 6 months after enhanced recovery after colorectal surgery

**DOI:** 10.1186/s13741-018-0099-2

**Published:** 2018-08-23

**Authors:** Thomas Deiss, Lee-lynn Chen, Ankit Sarin, Ramana K. Naidu

**Affiliations:** 1 Department of Biochemistry & Biophysics, University of California San Francisco, 505 Parnassus Ave., San Francisco, CA 94143 USA; 2 Department of Anesthesia & Perioperative Medicine, University of California San Francisco, 1825 4th St., San Francisco, CA 94158 USA; 3 Department of Surgery, University of California San Francisco, 1825 4th St., San Francisco, CA 94158 USA; 4 California Orthopedics and Spine, Director of Pain Management at Marin General Hospital, 18 Bon Air Road, Larkspur, CA 94939 USA

**Keywords:** Enhanced recovery after surgery, Colorectal surgery, Postoperative pain, Long-term outcomes, Hospital readmission

## Abstract

**Background:**

Enhanced recovery after surgery (ERAS) programs have been established as perioperative strategies associated with improved outcomes. However, intermediate and long-term patient-reported outcome data for patients undergoing ERAS interventions remain limited. We utilized an automated telephone survey 6 months post-colorectal surgery from patients who participated in an ERAS program to determine 6-month patient-reported outcomes and associated predictive factors.

**Methods:**

We conducted a prospective observational study, using an automated telephone survey and researcher-administered telephone questionnaire 6 months after patients underwent abdominal colorectal surgery. Six-month significant outcomes were defined by persistent pain, hospital readmission, and patient satisfaction. Patients reporting these outcome variables were compared with patients who met none of these criteria. Additionally, analysis was performed to determine differences between patients that did and did not respond to the 6-month survey. A chi-square test was used to determine any relationship for categorical variables, a two independent sample *t* test for length of procedure/stay, and a Wilcoxon-Mann-Whitney test for pain scores.

**Results:**

One hundred fifty-four of 324 patients contacted 6 months after surgery completed the automated telephone survey (47.53%). There was no statistical difference between patient populations completing and not completing the survey. Hospital 6-month readmission was associated with patients with a diagnosis of cancer (*P* = .049) and with a longer mean length of index procedure (282 vs. 206 minutes, *P* = .006). Median 6-month pain scores were higher for patients that underwent an open procedure compared to laparoscopic (*Z* = − 2.06, *P* = .04).

**Conclusions:**

Long-term benefits of an ERAS program were mostly confirmed. Longer procedure time and patients with cancer correlated with an increased likelihood of hospital 6-month readmission, suggesting that perioperative outcomes in complex cancer patients need to be evaluated over a longer time frame. In addition, invasiveness of procedure continues to have a significant effect on pain scores even 6 months later.

## Background

Enhanced recovery after surgery (ERAS) programs are becoming an essential component of any perioperative management paradigm. They have been shown to be effective at improving early postoperative outcomes in several settings (Lau and Chamberlain 2017; Visioni et al. 2017; Ni et al. 2015) and especially in colorectal surgery (Sarin et al. 2016a). However, information on recovery after discharge in this group of patients is limited (Jakobsson et al. 2014), and this information is becoming increasingly important as convalescence from surgery shifts to the outpatient setting in the era of shortening postoperative length of stays. Current postoperative data focuses on physiological parameters, which are key in the early postoperative period, but are neither available nor reflective of recovery in the post discharge longer term period when patients are under less surveillance by healthcare providers. Therefore, there is merit in obtaining and analyzing patient-reported outcomes during this period.

Previous studies (Kehlet et al. 2006a) have shown that acute postoperative pain is followed by persistent pain in 10–50% of individuals after common operations. Chronic postoperative pain can range from 15 to 30% after major and minor abdominal and pelvic operations, 6–36% following inguinal hernia, 3–56% following gall bladder surgery, (Perkins and Kehlet 2000), and 17% incidence for colorectal operations (Joris et al. 2015). In addition to pain, other factors such as fatigue, muscle weakness, and gastrointestinal dysfunction, which can persist for several weeks, contribute to the dissatisfaction felt by patients. Our study attempted to identify the percentage of patients that have a less than satisfactory 6-month outcome following colorectal surgery within an enhanced recovery program and to analyze the cause of these outcomes using patient-reported data.

## Methods

**Fig. 1:**
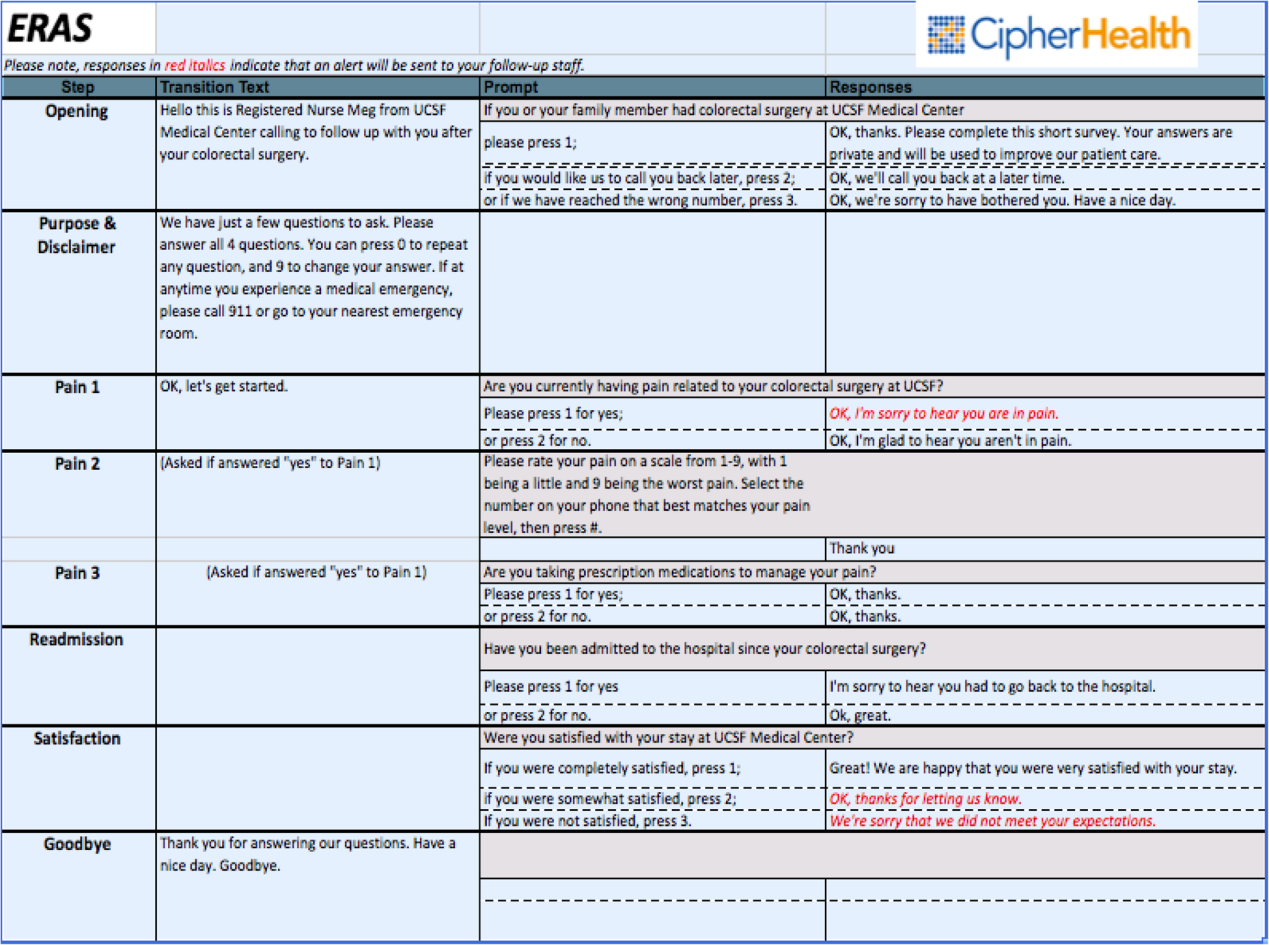
Script for automated telephone call

**Fig. 2:**
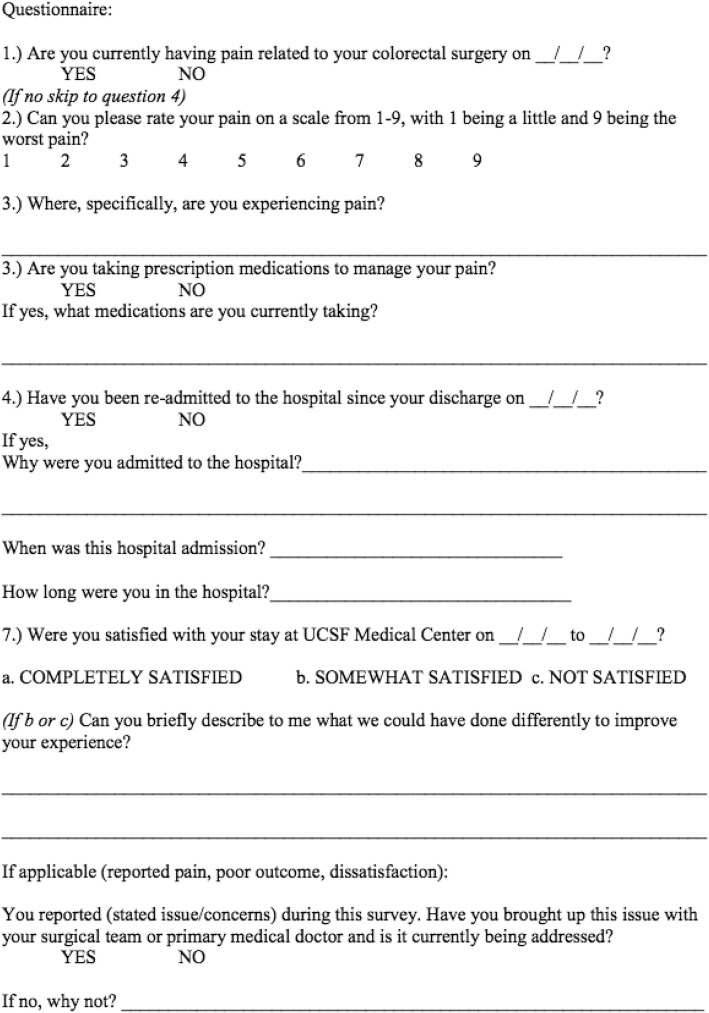
Research telephone questionnaire

After obtaining Institutional Review Board approval, we conducted a prospective observational study of patients that underwent abdominal colorectal surgery at a single tertiary medical center from February 2015 to June 2016. All adult patients (*n* = 324) that were part of the ERAS program for colorectal surgery within this period were included in the study. As part of standard care in the ERAS program, patients receive automated telephone calls (CipherHealth LLC, New York, NY) 6 months after discharge following surgery. The automated telephone call script was designed to ask questions about persistent pain, readmissions, and satisfaction with their stay at the medical center (Fig. 1). These data were collected monthly and sent to our research team. For the first 2 months of data collection, a researcher called patients that had completed the automated telephone call to validate their survey responses. The period of validation ended when no difference between the automated telephone call and research-administered questionnaire results were found. After the initial period of validation, a researcher called all patients that reported a significant outcome in the automated telephone call and administered a telephone questionnaire (Fig. 2). Significant outcomes were defined by any of the following: (1) pain associated with the surgery 6 months after discharge, (2) any hospital readmissions between discharge and 6 months, regardless of cause, and (3) patients reporting “somewhat satisfied” or “not satisfied” with their stay at the medical center. The researcher-administered telephone questionnaire confirmed data collected from the automated telephone call and also collected additional information. The additional information collected by the researcher-administered telephone questionnaire included, when applicable, the site of the patient’s pain, what specific medications were being taken for pain, when they were readmitted to a hospital, why they were readmitted to a hospital, the length of their hospital readmission, and/or what could have been done differently to improve their experience during their initial stay. Researcher-administered telephone questionnaires were done within 2 weeks of receiving the monthly data and up to three attempts were made to contact each patient. The Institutional Review Board granted a waiver of informed consent for patients in which only information from the medical record and automated telephone calls was obtained. For patients that completed the researcher-administered telephone questionnaire, verbal consent was first obtained for the collection of the questionnaire data.

Baseline patient and surgical data was retrieved from electronic medical records for all patients that received an automated telephone call 6 months post-colorectal surgery. Patient data included age, gender, diagnoses (cancer/non-cancer), and ASA rating. Surgical data included use of an epidural, type of procedure (open vs. minimally invasive), preoperative and postoperative pain scores, length of procedure, and length of hospital stay. Preoperative pain scores were collected in the pre-operative holding room when patients were checked in before surgery, and average postoperative pain scores were collected while patients were in the post-anesthesia care unit after surgery.

Significant outcomes were based off the six-month automated telephone call and researcher-administered telephone questionnaire. Patients reporting any of the significant outcomes were compared with patients that did not report any significant outcomes.

Data were analyzed using STATA software (Version 12.1, StataCorp LLC, College Station, TX). Statistical analysis was performed to determine any relationship between surgical or patient data and the significant outcomes. *P* values were obtained from the chi-square test to determine any relationship between the categorical variables (age, gender, diagnoses, ASA rating, type of procedure, epidural use) and each significant outcome. *P* values were obtained from the two independent sample *t* test to determine the relationship between length of procedure and length of stay to each significant outcome. A Wilcoxon-Mann-Whitney test was used to determine any relationship between preoperative and postoperative pain (1–10 scale used for both) and any significant outcome. Analysis was also performed to determine if there was a statistically significant difference between the surgical and patient data of those who completed the automated 6-month survey and those that did not, using the same statistical tests described above (Table 1).

**Table 1:** Difference between the patient population that completed the automated 6-month telephone survey and the population that did not complete the survey

	Completed 6-month call (*N* = 154)	Unsuccessful 6-month call (*N* = 170)	*P* value
Age	55 ± 14	53 ± 15	0.246
Male sex	81 (53)	80 (47)	0.319
Dx of cancer	75 (49)	73 (43)	0.299
Open procedure	64 (42)	73 (43)	0.801
Median length of stay (hours)	126	126	0.839
Median length of Procedure (min)	187	197	0.572
Median ASA rating	2	2	0.579
Pre-op pain	29 (19)	40 (24)	0.302
Post-op pain	73 (47)	81 (48)	0.948
Epidural use	90 (58)	108 (64)	0.348

## Results

**Fig. 3:**
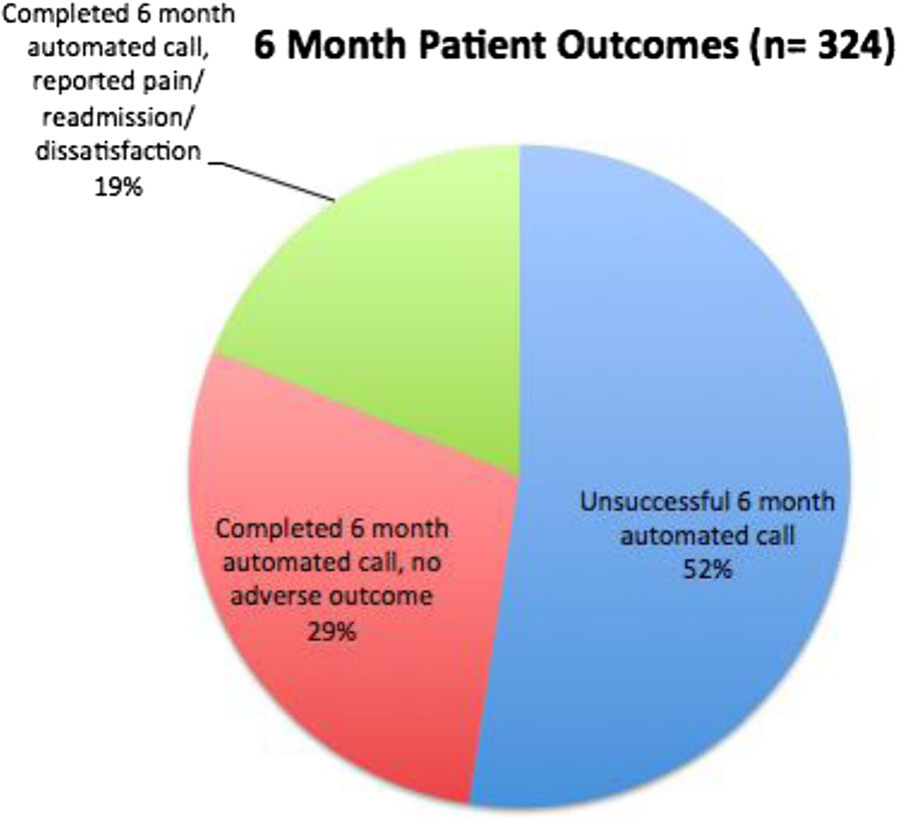
Six-month patient outcomes based on automated call data

During the 17-month study period from February 2015 to June 2016, 324 patients received automated telephone calls 6 months after discharge following colorectal surgery. One hundred fifty-four of 324 (48%) patients completed the automated telephone survey and 39 of 324 (12%) patients completed the researcher-administered telephone questionnaire. Of the patients that completed the 6-month automated telephone call, 61 of 154 (40%) reported a significant outcome (Fig. 3). Twenty-seven of those 61 patients (44%) completed a researcher-administered telephone questionnaire, while the remaining 34 patients (56%) could not be reached in the follow-up call and/or all relevant data was collected from the automated telephone questionnaire and electronic medical records at UC San Francisco Medical Center. One patient that was contacted by a researcher refused participation in the telephone questionnaire, and none of that patient’s data was included in the study. There were no statistical differences between those completing and not completing the telephone survey when examining age, gender, cancer diagnosis, and variables related to the patient’s procedure (Table 1).

**Fig. 4:**
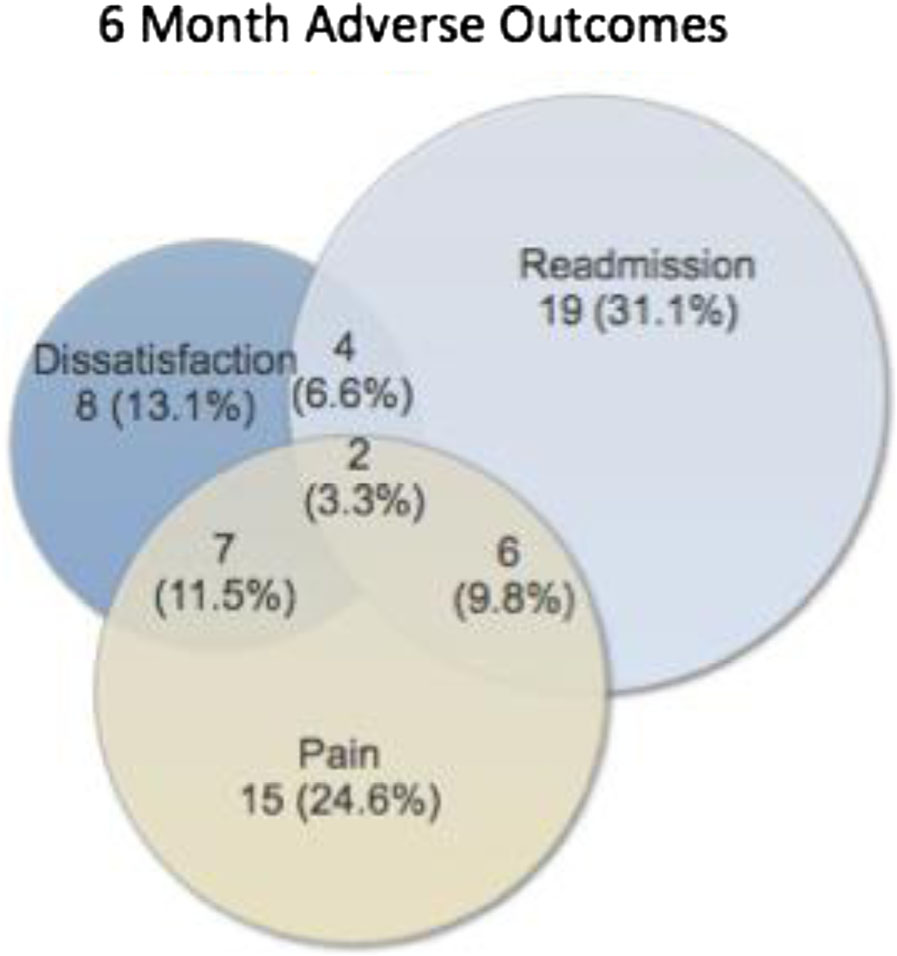
Breakdown of 6-month patient-reported outcomes

Thirty of 154 (19%) patients reported persistent surgical pain, 31 of 154 (20%) patients reported a hospital readmission, and 21 of 154 (14%) patients reported less than complete satisfaction with their stay (Fig. 4).

Of the 30 patients reporting persistent surgical pain, 19 (63%) reported taking medication for their pain, 10 of which were using opioids to manage their pain. Median 6-month pain scores were significantly higher for patients that underwent an open procedure compared to minimally invasive (*Z* = − 2.06, *P* = 0.04). All patients reporting persistent pain confirmed that the site of pain was surgical and not a chronic or non-surgical pain.

Of the patients reporting less than complete satisfaction with their hospital stay, postoperative pain (9 of 21, 43%) and postoperative complications (8 of 21, 38%) were the most common reasons.

Hospital readmission was associated with a diagnosis of cancer (*P* = .049) and with longer mean length of procedure (282 vs. 206 min, *P* = .006) (Table 2). Of the patients with hospital readmissions, readmission data was only available for 22 of 31 (71%) patients. Of those patients, 10 of 22 (45%) readmissions occurred within 30 days of discharge and 8 of 22 (36%) readmissions occurred over 90 days after discharge. The most common reason for readmission was bowel obstruction in 3 of 22 (14%) patients.

**Table 2:** Characteristics of patients with and without a hospital readmission within 6 months of discharge

	Hospital readmission within 6 months of discharge (*N* = 31)	No hospital readmission within 6 months of discharge (*N* = 123)	*P* value
Age	57 ± 16	54 ± 14	0.290
Male sex	21 (68)	60 (49)	0.059
Dx of cancer	20 (65)	55 (45)	0.049
Open procedure	12 (39)	52 (42)	0.719
Length of stay (hours)	202 ± 118	184 ± 170	0.839
Length of procedure (min)	282 ± 167	206 ± 126	0.006
Median ASA rating	2	2	0.127
Pre-op pain	7 (23)	22 (18)	0.550
Post-op pain	14 (48)	61 (50)	0.836
Epidural use	19 (61)	71 (58)	0.719
Dissatisfaction with stay	6 (19)	15 (12)	0.299
6 month pain	8 (26)	22 (18)	0.320

No relationship was identified between preoperative pain and 6-month significant outcomes nor postoperative pain (in the post-anesthesia care unit) and 6-month significant outcomes. Examining pain trajectories for patients, there was no significant correlation with pre- or postoperative pain and 6-month reported pain.

## Discussion

The purpose of this observational study was to assess patient-reported outcomes 6 months after participation in an enhanced recovery after colorectal surgery program. Besides the immediate benefits of reduction in length of stay and cost, it is important to understand whether such an ERAS program could improve longer-term postoperative outcomes such as patient satisfaction, readmissions, and chronic pain. Hospitals typically use surveys from vendors such as Press Ganey to collect patient satisfaction data after an inpatient stay (between 48 h and 6 weeks after discharge). With our initial rollout of ERAS for the colorectal service line, we observed improved patient outcomes while maintaining our Press Ganey scores (Sarin et al. 2016b). However, most studies and registries have concentrated on 30-day outcomes.

By using a similar automated phone call system to screen for patient-reported post-operative pain, readmission, and/or satisfaction, we were able to focus on 6-month outcomes without requiring significant additional resources.

The survey response rate of 48% compares favorably with results reported in the literature (Kehlet et al. 2006b). Since there was no statistical difference between the responders and non-responders to the survey (based on age, gender, cancer diagnosis, and variables related to the patient’s procedure), it was assumed that the reported outcomes for responders would represent those of non-responders. Similar survey techniques can be considered in the future.

Though the majority of patients were satisfied with their surgical experience, those who were dissatisfied either suffered from persistent post-surgical pain or another complication related to their surgery. These longer term patient-reported outcomes are often underappreciated by the perioperative team. Our study suggests that perioperative complications and persistent pain can be a source of patient dissatisfaction long after the index surgery when they may no longer be under the care of their surgeon.

The development of chronic persistent post-surgical pain (PPSP) is an area of growing interest. It is estimated that the incidence of PPSP after abdominal surgery is anywhere from 10 to 50% depending on the type of surgery (cholecystectomy, herniorrhaphy, laparotomy) and surveying methods (Kehlet et al. 2006b; Laufenberg-Feldmann et al. 2016). One of the main objectives of this study was to determine the prevalence of PPSP in patients after colorectal surgery and understand the role of the ERAS program in affecting this pain. This study showed a PPSP rate of 19%, of which 63% of patients were taking pain medications to address this pain. Of note, only 33% of patients were using opioids to manage this pain, which we hypothesize is directly related to their participation in an ERAS program focused on multimodal analgesia and patient education on attenuating opioid consumption postoperatively. With the current American opioid epidemic, this is a reassuring outcome.

Readmission rates at 30 days were 10%, which is similar to previously published data. The majority of readmissions were related to infection, obstruction, or nausea/vomiting. Not all data regarding readmissions were available, because patients might have been admitted to other institutions. Readmission rate at 6 months was 20%, which was twice the readmission rate at 30 days. Readmissions were associated with a diagnosis of cancer and with longer surgical procedure times, suggesting that patients with more complex operations or underlying malignancy were driving longer term readmissions, rather than postoperative care.

### Limitations

This is an observational study and is dependent on telephone responses; therefore, it is inherently susceptible to selection bias. Detailed readmission data was only available for patients admitted to our institution, although the telephone surveys did capture all readmissions (subject to recall bias) as these were reported by patients themselves. The vendor through which the automated telephone surveys were administered only offered a 9-point scale for pain scores, rather than the standard 11-point numerical rating scale (NRS), which may have introduced slight differences in pain score results.

## Conclusion

Nineteen percent of patients after colorectal surgery within an ERAS program went on to develop persistent post-surgical pain. However, only two out of three used any analgesic medications, and only one out of three used opioids to mitigate this pain, suggesting that a focus on multimodal non-opioid analgesia in the ERAS program may be of benefit beyond just the immediate postoperative period. Pain and postoperative complications account for the majority of dissatisfaction in patients 6 months following colorectal surgery. Readmissions occur as often in the period from 1 to 6 months following surgery as they do in the first month, and this is related to more complex surgical procedures and a diagnosis of cancer.

## Abbreviations

ASA: American Society of Anesthesiologists
ERAS: Enhanced recovery after surgery
PSPP: Persistent post-surgical pain
